# Prevalence of congenital microcephaly and its risk factors in an area at risk of Zika outbreaks

**DOI:** 10.1186/s12884-021-03705-9

**Published:** 2021-03-17

**Authors:** Songying Shen, Wanqing Xiao, Lifang Zhang, Jinhua Lu, Anna Funk, Jianrong He, Si Tu, Jia Yu, Li Yang, Arnaud Fontanet, Wei Bao, Kar Keung Cheng, Xiu Qiu

**Affiliations:** 1 Division of Birth Cohort Study, Guangzhou Women and Children’s Medical Center, Guangzhou Medical University, Guangzhou, 510623 China; 2 Guangdong Provincial Key Clinical Specialty of Woman and Child Health, Guangzhou, 510623 China; 3 Department of Pediatrics, University of Calgary, Calgary, T2N 1N4 Canada; 4 Emerging Disease Epidemiology Unit, Institut Pasteur, 75015 Paris, France; 5 Department of Epidemiology, College of Public Health, University of Iowa, Iowa City, Iowa, 52242 USA; 6 Institute of Applied Health Research, College of Medical and Dental Sciences, University of Birmingham, B15 2TT, Birmingham, UK; 7 Guangdong Provincial Clinical Research Center for Child Health, Guangzhou, 510623 China

**Keywords:** Microcephaly, Prevalence, Risk factor, Preterm birth, Small for gestational age

## Abstract

**Background:**

Prevalence of neonatal microcephaly in populations without Zika-epidemics is sparse. The study aimed to report baseline prevalence of congenital microcephaly and its relationship with prenatal factors in an area at risk of Zika outbreak.

**Methods:**

This study included singletons born after 24 gestational weeks in 2017–2018 at four hospitals in Guangzhou, China. Microcephaly was defined as a head circumference at birth >3SD below the mean for sex and gestational age. Prevalence of microcephaly was estimated by binomial exact method. Multivariable logistic regression was used to examine the associations of microcephaly with prenatal factors. The population attributable fraction (PAF) for associated risk factors was calculated.

**Results:**

Of 46,610 live births included, 154 (3.3, 95% CI 2.8–3.9 per 1000 live births) microcephalies were identified. Maternal hepatitis B virus carriers (HBV, OR 1.80, 95% CI 1.05–3.10) and primipara (OR 2.68, 95% CI 1.89–3.81) had higher risk of having a microcephalic baby. Higher prevalence of microcephaly was observed in women who had premature labor (OR 1.98, 95% CI 1.17–3.34) and had a baby with fetal growth restriction (OR 16.38, 95% CI 11.81–22.71). Four identified factors (HBV, primiparity, preterm labor, and fetal growth restriction) contributed to 66.4% of the risk of microcephaly.

**Conclusions:**

The prevalence of microcephaly in Guangzhou was higher than expected. This study identified four prenatal risk factors that, together, contributed to two-thirds of the increased risk of microcephaly. This is the first reported association between maternal HBV carrier status and microcephaly.

## Background

Congenital microcephaly is characterized as a smaller head compared to others of the same sex and gestational age. Infants with microcephaly have a significantly increased risk of developmental delay, intellectual disability, long-term disability, and even mortality [1–5]. The World Health Organization (WHO) recommends that early intervention with multidisciplinary approaches should be used to promote the neurodevelopment of babies with microcephaly [6]. Early intervention in infants with neurodevelopmental disorders that focus on reducing problems and maximizing a child’s abilities can help improve the children’s quality of life [7]. An understanding of the maternal and neonatal factors related to microcephaly at birth is likely to facilitate early identification of microcephaly and thus effective intervention.

Relatively well-known causes of microcephaly include maternal infections, such as Zika virus and cytomegalovirus, genetic factors, and teratogens. These understandings are mainly based on evidence from recent Zika-epidemic areas [8–10]. The epidemiology of microcephaly in populations without an outbreak of Zika virus infection is poorly described. In addition, congenital microcephaly can be subdivided according to the proportionality related to the overall anthropometry [11, 12]. Proportionate congenital microcephaly might result from an intrauterine dystrophy [11] that impacts both head and body growth, whereas disproportionate microcephaly presents head growth lagged behind somatic growth or weight gain [12]. Case series of pregnant women with ZIKV infections have reported a disproportionate fetal growth profile [13–15], especially in those infected in early stage of pregnancy [13], the mechanism of which has been revealed by a recent animal study [16]. The proportionality of head to body size is also proposed to improve the classification and prognosis of microcephaly in clinic [11, 17, 18]. In spite of the evidence above, the epidemiology of different subtypes of microcephaly remains largely unknown.

Guangzhou, located in South China, is an area with frequent epidemics of dengue virus, which is a flavivirus of the same genus as Zika. It is possible that maternal immunity to dengue virus promotes Zika infection and Zika virus–induced microcephaly in fetuses [16]. Its natural environment is also facilitative for Zika transmission. Meanwhile, as a cosmopolitan city and an important hub port city of “the Belt and Road” in South China, Guangzhou shares frequent communications with countries where Zika virus is circulating, which may also facilitate the transmission of the disease. Evidence from Americas [19, 20] and a recent study from Angola [21] showed that prolonged local transmission of Zika virus may have existed before the detection of the outbreak, highlighting the challenge for potential risk of Zika transmission faced by South China. Improved understanding of the baseline epidemiology of microcephaly in such areas is essential to evaluate the true severity of an eventual outbreak. Using the data from a large cross-sectional study in Guangzhou, China, we aimed to estimate the prevalence of, and perinatal factors related to congenital microcephaly and its subtypes.

## Methods

### Study design, setting and population

As head circumference (HC) measurement is not routinely collected and recorded in clinical practice in China, a surveillance study for HC at birth was introduced in February 2017 in four hospitals in Guangzhou, China. The four hospitals were selected using a cluster sampling from 13 municipal and district-level Maternal and Child Care Service Centers in Guangzhou, including two municipal tertiary healthcare centers (two campuses of Guangzhou Women and Children’s Medical Center), one district-level tertiary centers (Huadu Maternal and Child Care Service Center) and one district-level secondary centers (Liwan Maternal and Child Care Service Center), located in the central, north and west areas of Guangzhou, China, respectively [22]. GWCMC provides services to pregnancies from all 11 districts of the city. Huadu and Liwan Maternal and Child Care Service Center mainly provide services to pregnancies from Huadu and Liwan district, respectively. The number of live births in these four study hospitals accounted for 14.1% of total live births in Guangzhou city during the study period. Singletons born at 24–42 gestational weeks between February 10th, 2017 and May 31st, 2018 were included. Stillbirths and those with a brain and central nervous system (CNS) abnormality (ICD-10 code: O35.0, Q00-Q07 excluding Q02), and/or chromosome abnormality (ICD-10 code: O351, Q90-Q99), with unknown sex, or with missing or implausible data on HC were excluded. The study protocol was approved by the Guangzhou Women and Children’s Medical Center Ethics Approval Board (No. 2016111865–2).

### HC measurements and definition of microcephaly

The HC of the newborns was measured at each study center by a uniform instrument, namely the Seca HC measuring band 212, made of non-stretching Teflon. The midwives were trained to follow a standard protocol before the study was started. The definition of HC is the widest possible circumference of the head around the back of the head with the measuring band held above eyebrow and ears. The HC measurements of the newborns were completed immediately after birth. All measurements were read to the nearest millimeter and then recorded. Each newborn was measured twice and the average value was recorded. The maximum difference accepted between the two measurements was 4 mm.

The Z-scores of HC at birth was calculated according to INTERGROWTH-21st newborn size standards by gestational age and sex [23]. Microcephaly was defined as an HC Z-score at birth < − 3.0 [6, 11, 24]. According to the HC-to-birth weight proportionality, microcephaly was grouped into two subtypes: disproportionate microcephaly, defined as the difference between HC Z-score and birth weight Z-score (namely HC Z-score minus birth weight Z-score) < − 3.0, which represents a head growth being lagged behind body growth by 3 Z-score when refering to the standard population; proportionate microcephaly, defined as the difference between HC Z-score and birth weight Z-score ≥ − 3.0.

### Data abstraction

Data on maternal age, parity, gestational diabetes mellitus (GDM), hypertensive disorders of pregnancy (HDP), birth weight, gestational age at delivery, and newborn sex were obtained from the Guangzhou Perinatal Health Care and Delivery Surveillance System, which covers more than 99% of deliveries in Guangzhou [25]. GDM diagnosis was based on the International Association of Diabetes and Pregnancy Study Groups criteria (IADPSG criteria, FPG ≥ 5.1 mmol/l, 1 h glucose≥10.0 mmol/l, and 2 h glucose≥8.5 mmol/l) [26]. HDP included pre-existing hypertension with or without superimposed proteinuria, gestational hypertension without significant proteinuria, preeclampsia, and eclampsia [27]. All pregnant women were screened for serum HBsAg at their first antenatal visit in GWCMC using enzyme-linked immunosorbent assay (ELISA) kits (Shanghai Kehua bio-engineering Co., Ltd., China). Maternal carrier of hepatitis B virus (HBV) was defined as positive HBs Ag. All neonates of mothers with positive HBs Ag received hepatitis B vaccine and hepatitis B immune globulin immediately after birth and hepatitis B vaccine at 1 month and 6 months of age. Because an electronic medical record system is only established in GWCMC, data on maternal carriers of HBV (ICD-10 code: Z22.5) and maternal hepatitis B were only available and obtained from the electronic medical records in two GWCMC campuses through linkage to the hospital information system with a unique identifier.

Birth weight was measured by midwives immediately after delivery. Birth weight Z-scores were calculated according to INTERGROWTH-21st newborn size standards by gestational age and sex [23]. Gestational age was estimated from ultrasound examination during the first or second trimester. Fetal growth restriction (FGR) was defined as a birth weight lower than the 10th percentile for gestational age by sex. Preterm labor was defined as labor before 37 weeks of gestation.

### Statistical analysis

Robust regression with the iteratively reweighted least square procedure was used to identify implausible HC values either caused by misclassification of gestational age or by invalid HC measurements. HC values with residual out of range of − 3.89 SD and + 3.89 SD were considered as outliers [28, 29].

The prevalence of microcephaly and its subtypes was estimated and the 95% CI was calculated using the binomial exact method. Data were summarized as means (standard deviations) for continuous variables and frequency (percentage) for categorical variables.

Multivariable logistic regression models were applied to estimate the unadjusted and adjusted odds ratios (ORs) and 95% confidence intervals (CIs) for the association between prenatal characteristics and the risk of microcephaly or microcephaly subtypes, with non-microcephaly (HC Z-score at birth ≥ − 3.0) as the reference. In models for the associations with maternal age, GDM, HDP, and parity, these characteristics entered the model simultaneously and were mutually adjusted for each other. The association with maternal carrier of HBV was assessed among births in GWCMC, adjusted for maternal age at conception, parity, HDP, GDM, year of birth, and place of birth. Based on the year when routine hepatitis B immunization for newborns was implemented, the year of birth was grouped into a binary variable (“before 1992” and “in 1992 or after”) [30, 31]. Maternal place of birth was classified into two regions according the distribution of HBV infection, including the eastern region (including Beijing, Fujian, Guangdong, Jiangsu, Liaoning, Shandong, Shanghai, Tianjin, and Zhejiang) and the central/western region (including Anhui, Hainan, Hebei, Heilongjiang, Henan, Hubei, Hunan, Jiangxi, Jilin, Shanxi, Chongqing, Gansu, Guangxi, Guizhou, Inner Mongolia, Ningxia, Qinghai, Shaanxi, Sichuan, Tibet, Yunnan, and Xinjiang) [32]. In the models to examine the association of microcephaly with preterm labor and FGR, maternal age, parity, HDP, GDM, and infant’s sex were adjusted for. We calculated the population attributable fraction (PAF) and its 95% confidence interval associated with identified factors for the risk of microcephaly and its subtypes in the study population, using a published %PAR SAS macro [33].

All analyses were done with SAS version 9.3 (SAS Institute, Cary, NC, USA). *P* < 0.05 was considered significant.

## Results

A total of 47,369 singletons were eligible, with 17,798 births from Zhujiang Newtown Campus of GWCMC, 12,270 births from Maternal-Infant Campus of GWCMC, 13,217 from Guangzhou Huadu Maternal and Child Care Service Center, and 4084 from Guangzhou Liwan Maternal and Child Care Service Center. After excluding newborns with unknown sex (*n* = 3) and missing (*n* = 328) or implausible HC data (*n* = 145), stillbirths (*n* = 222), and those with brain and central nervous system (CNS) abnormalities (*n* = 25) and/or chromosome abnormalities (*n* = 36), 46,610 newborns were included in the final analysis.

There were 154 newborns identified with microcephaly across all births, with a prevalence of 3.3 (95% CI, 2.8–3.9 per 1000 live births). Of these, 14 were disproportionate, with a prevalence of 0.3 (95% CI, 0.2–0.5 per 1000 live births), and the remaining 140 were proportionate, with a prevalence of 3 (95% CI, 2.5–3.5 per 1000 live births).

Table 1 shows the characteristics of the study population. There were 83.7% (39, 019/46,610) of mothers who were less than 35 years of age. The proportion of mothers who were primiparous was 41.9% (19,534/46,610). There were 3.2% (1493/46,610) and 14.6% (6787/46,610) of mothers diagnosed with HDP and GDM, respectively. A total of 7.5% (2189/29,187) of mothers were carriers of HBV, of whom only 9 were diagnosed with hepatitis B. The proportions of preterm labor and FGR were 5.6 and 7.0%, respectively. Male accounted for 53.5% (*n* = 24,927) of the newborns. The mean HC Z score, birth weight Z score and difference between the HC Z-score and birth weight Z-score were − 0.3 (SD 1.0), 0.0 (SD 0.9), and 0.3 (0.8), respectively.

**Table 1:** Characteristics of 46,610 mother-child dyads

Characteristics	Mean (SD)	n (%)
Maternal age	30.2 (4.7)	
< 35 years old		39,019 (83.7)
Primiparous		19,534 (41.9)
Hypertensive disorders of pregnancy		1493 (3.2)
Gestational diabetes		6787 (14.6)
Maternal carrier of HBV^a^		2189 (7.5)
Sex of newborns (Male)		24,927 (53.5)
Gestational age at birth, weeks	39.0 (1.5)	
Preterm birth		2590 (5.6)
Birth weight of newborns, kg	3.2 (0.4)	
Birth weight for gestational age of newborns, Z-score^b^	0.0 (0.9)	
Small for gestational age^c^		3265 (7.0)
Head circumference of newborns, cm	33.3 (1.3)	
Head circumference of newborns, Z-score^b^	−0.3 (1.0)	
< −3.0		154 (0.3)
≥ −3.0 & < −2.0		1777 (3.8)
BWHC Z-score difference^d^	−0.3 (0.8)	
< −3.0		58 (0.1)
≥ −3.0 & < −2.0		987 (2.1)

*HBV* Hepatitis B Virus, *SD* Standard deviation, *BWHC* Birth weight and head circumference of newborns

^a^Analyzed among the births born in two campuses of Guangzhou Women and Children’s Medical Center (*n* = 29,493)

^b^Calculated according to the INTERGROWTH-21st standards

^c^Defined as gestational age and sex-adjusted birth weight < 10th percentile based on the INTERGROWTH-21st newborn size standard

^d^Defined as the difference between head circumference Z-score and birth weight Z-score of newborns

**Fig. 1:**
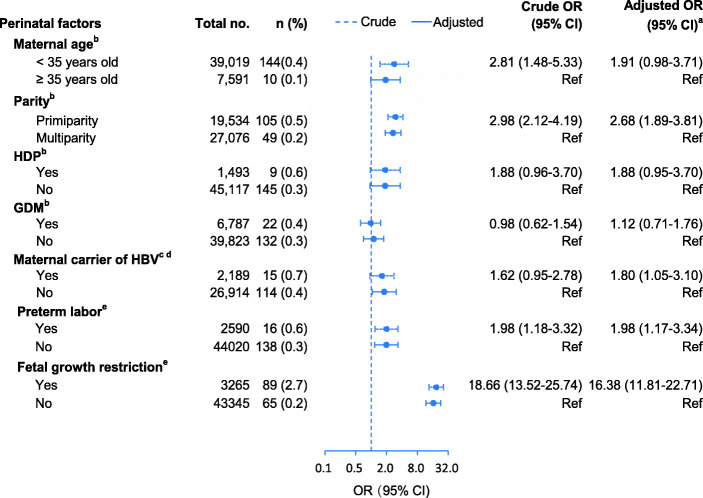
Pernatal factors associated with microcephaly. OR, odds ratio; HDP, hypertensive disorders of pregnancy; GDM, Gestational diabetes mellitus; HBV, Hepatitis B Virus. ^a^Non-microcephaly (head circumference Z-score ≥ − 3.0) as the reference group. ^b^In models for the associations with maternal age, GDM, HDP, and parity, these characteristics entered the model simultaneously and were mutually adjusted for each other. ^c^Analyzed only among all births born in two campuses of Guangzhou Women and Children’s Medical Center. ^d^Adjusted for maternal age, hypertensive disorders of pregnancy, and gestational diabetes mellitus and parity, year of birth, and place of birth. ^e^Adjusted for maternal age, hypertensive disorders of pregnancy, and gestational diabetes mellitus, parity and sex of newborn.

Associations between prenatal factors and microcephaly are shown in Fig. 1. Being a maternal carrier of HBV (adjusted OR 1.80; 95% CI, 1.05–3.10) and primiparity (adjusted OR 2.68; 95% CI, 1.89–3.81) were significantly associated with microcephaly. There was no association of microcephaly with maternal age, GDM, or HDP. Higher microcephaly risk was associated with preterm birth (OR 1.98; 95% CI, 1.17–3.34), SGA (OR 16.38; 95% CI, 11.81–22.71).

Associations between prenatal factors and microcephaly subtypes are shown in Table 2. Newborns had a higher risk of proportionate microcephaly if they were SGA (adjusted OR 20.76, 95% CI 14.61–29.51) or if their mothers were primiparous (adjusted OR 2.83, 95% CI 1.95–4.10) or had HDP (adjusted OR 2.06, 95% CI 1.04–4.07). Newborns who were born preterm (adjusted OR 14.24, 95% CI 4.91–41.30) had a higher risk of disproportionate microcephaly. Maternal HBV carrier status was not significantly associated with either proportionate (adjusted OR 1.73 95% CI 0.97–3.09) or disproportionate (adjusted OR 2.51, 95% CI 0.55–11.53) microcephaly.

**Table 2:** Perinatal factors associated with microcephaly phenotypes

Perinatal factors	Disproportionate microcephaly		Proportionate microcephaly	
	n (%)	Adjusted OR (95% CI)^a^	n (%)	Adjusted OR (95% CI)^a^
Maternal age^b^				
< 35 years old	13 (0.03)	1.90 (0.23–15.42)	131 (0.34)	1.91 (0.95–3.85)
≥ 35 years old	1 (0.01)	Ref	9 (0.12)	Ref
Parity^b^				
Primiparity	8 (0.04)	1.64 (0.55–4.87)	97 (0.50)	2.83 (1.95–4.10)
Multiparity	6 (0.02)	Ref	43 (0.16)	Ref
HDP^b^				
Yes	0 (0.00)	–	9 (0.60)	2.06 (1.04–4.07)
No	14 (0.03)	–	131 (0.29)	Ref
GDM^b^				
Yes	1 (0.01)	0.52 (0.07–4.04)	21 (0.31)	1.18 (0.74–1.89)
No	13 (0.03)	Ref	119 (0.30)	Ref
Maternal carrier of HBV^c, d^				
Yes	2 (0.09)	2.51 (0.55–11.53)	13 (0.59)	1.73 (0.97–3.09)
No	10 (0.04)	Ref	104 (0.39)	Ref
Preterm birth^e^				
Yes	6 (0.23)	14.24 (4.91–41.30)	10 (0.39)	1.29 (0.68–2.47)
No	8 (0.02)	Ref	130 (0.30)	Ref
Small for gestational age^e^				
Yes	0 (0.00)	–	89 (2.73)	20.76 (14.61–29.51)
No	14 (0.03)	–	51 (0.12)	Ref

*OR* Odds ratio, *CI* Confidence interval, *HDP* Hypertensive disorders of pregnancy, *GDM* Gestational diabetes mellitus, *HBV* Hepatitis B Virus

^a^Non-microcephaly (head circumference Z-score ≥ −3.0) as the reference group

^b^In models for the associations with maternal age, GDM, HDP, and parity, these characteristics entered the model simultaneously and were mutually adjusted for each other

^c^Analyzed only among all births born in two campuses of Guangzhou Women and Children’s Medical Center

^d^Adjusted for maternal age, hypertensive disorders of pregnancy, and gestational diabetes mellitus and parity, year of birth, and place of birth

^e^Adjusted for maternal age, hypertensive disorders of pregnancy, gestational diabetes mellitus, and parity, sex of newborn

The PAF associated with identified risk factors for microcephaly and its subtypes are presented in Table 3. In the study population, 66.4% (95% CI 43.9–81.1%) of the risk of microcephaly could be attributed to the four associated risk factors, including primiparity, maternal HBV carriage status, SGA, and preterm birth. The most important factors were SGA and primiparity, with PAF being 50.6% (95% CI 38.8–60.8%) and 28.9% (95% CI 10.3–45.5%), respectively. For disproportionate microcephaly, the PAF associated with preterm birth was 47.5% (95% CI 13.1–71.7%). For proportionate microcephaly, 56.5, 28.2, and 1.9% of the risk could be attributed to SGA, primiparity, and HDP, respectively, constituting a combined PAF of 67.7% (95% CI 47.7–81.1%).

**Table 3:** Population attributable fractions and 95% confidence interval associated with identified factors for microcephaly and its subtypes

Risk factors	Microcephaly	Disproportionate microcephaly	Proportionate microcephaly
All risk factors combined	66.4% (43.9–81.1%)	47.5% (13.1–71.7%)	67.7% (47.7–81.1%)
Specific factors			
Primiparity	28.9% (10.3–45.5%)	–	28.2% (7.9–46.3%)
HDP	–	–	1.9% (− 3.2 to 6.9%)
Maternal carrier of HBV	4.8% (−1.2 to 10.8%)	–	–
Small for gestational age	50.6% (38.8–60.8%)	–	56.5% (44.0–66.9%)
Preterm birth	4.0% (− 1.4 to 9.4%)	47.5% (13.1–71.7%)	–

*HDP* Hypertensive disorders of pregnancy, *HBV* Hepatitis B Virus

## Discussion

This multi-center study found that the prevalence of microcephaly was 0.33%, which is higher than the expected risk of 0.13% in a population with normally distributed HC and using a definition of HC Z score smaller than − 3. Microcephaly was associated with maternal HBV carrier status, primiparity, FGR, and preterm birth, which together contributed to two-thirds of the risk of microcephaly. The risk factors associated with microcephaly varied between proportionate and disproportionate microcephaly.

Although the estimated prevalence of microcephaly in the present study is lower than pre-Zika virus epidemic estimate in two cities in Brazil (5 ~ 7 per 1000 births), where the diagnosis of microcephaly was also based on the INTERGROWTH-21st standard [34], it was ~ 2 folds higher than the expected rate (1.3 per 1000 births). The rate is also much higher than that from birth defect surveillance systems in other regions of the world, which were reported as 0.20 per 1000 births in Europe, 0.23 per 1000 births in India, 0.44 per 10 00 births in South America pre-Zika, and around 0.87 per 1000 live births before the Zika virus epidemic in the US [17, 35–37]. The variation in the definition of microcephaly used is likely to contribute largely to the variation in the reported prevalence across the studies mentioned. Nevertheless, this study showed that microcephaly is sub-endemic in a region without ongoing Zika virus transmission.

Considering that early neurodevelopmental interventions recommended by the World Health Organization could improve microcephalic children’s quality of life [6], identification of risk factors and comorbidities for microcephaly and its subtypes may allow for timely diagnoses and enable clinical practitioners to provide appropriate counseling about the long-term prognosis. The present study adds to the evidence that risk factors associated with microcephaly varied between two subtypes, proportionate and disproportionate, of microcephaly.

In the present study, we found that infants with proportionate microcephaly included more infants with SGA, 18–22% of whom were reported constitutionally small but healthy in the previous studies [38]. As the definition of microcephaly was only based on head circumference rather than aetiological investigations, it is inevitable that infants with proportionate microcephaly include some SGA infants whose head sizes are small but without structural brain lesion [39].

The definition of disproportionate microcephaly in the present study represents a head growth being significantly lagged behind body growth. Previous studies defined disproportionate microcephaly as HC Z-score < − 3 and weight or height Z-score ≥ − 3, which will include a large portion of infants whose head growth being little lagged behind body growth [12, 34]. It is intriguing to find that disproportionate microcephaly is associated with preterm labor, especially as preterm labor has itself been linked to neurodevelopmental delays [40–43]. On one hand, the finding may suggest that factors contributing to preterm labor might also have an impact on those related to the pathogenesis of microcephaly, such as neural progenitor cell proliferation, differentiation, and apoptosis [8]. Further studies are warranted for identifying these factors. On the other hand, the disruption of the normal intrauterine neurodevelopment, a critical period for brain growth and maturation, would further exacerbate the neurodevelopmental disorders of the children [40, 43]. Thus, disproportionate microcephaly may have a greater chance of representing ‘true’ microcephaly. Identification of disproportionate microcephaly in preterm babies and referring them to early intervention services to improve neurodevelopmental outcomes would be urgently needed.

This study is the first to report the association between maternal HBV carrier status and a higher risk of microcephaly. Previous studies regarding maternal virus infection and microcephaly focused mainly on the Zika virus and were conducted mainly in Zika-epidemic areas. Some previous studies have found that Hepatitis B surface antigen (HBsAg) positivity during pregnancy increased the risk of congenital malformation [44]. However, few studies have investigated the influence of maternal HBsAg positivity on fetal brain growth. As TORCH (Toxoplasma gondii, other microorganisms, rubella virus, cytomegalovirus, and herpes simplex virus) infections are one of the main causes of microcephaly, future studies are needed to confirm this association and determine the public health impact of maternal HBV infection on the risk of microcephaly in regions with high HBV infection burden, such as in China and Africa [45].

Major strengths of this study include the large sample size, which allows for the identification of novel risk factors of conditions with low prevalence, and the high-quality of HC data that was ensured by standardized measurements. Importantly, using high quality data we provide approximate population-based baseline prevalence estimations for future surveillance, which will become very important in the case of outbreaks of Zika or other infectious diseases in this region of China. This study has some limitations. First, we only collected the data in one developed city in China, which limits its generalization to other regions. However, Guangzhou is a megalopolis with a large migrating population moving in from other areas of China. Second, the association between HBV and microcephaly should be interpreted with caution because potential unrecognized confounders within populations with higher hepatitis B rates may drive this association. However, in a subgroup of pregnant women who participated in both the current study and the Born in Guangzhou Cohort Study [46], the ORs changed very little after additional adjustment for known influential factors, such as alcohol consumption, smoking, and use of emergency contraceptives during early pregnancy (data not shown). This suggests that the observed associations are unlikely to be largely changed by adjustment for known confounders. Third, the power to detect the associations between risk factors and disproportionate microcephaly might be limited due to the small sample size of this subgroup. Finally, only microcephaly-status and comorbidities at birth were observed. Long-term neurodevelopmental and other outcomes of infants with microcephaly, according to subtype, at birth should be further evaluated in future studies in order to better understand the clinical significance of finding microcephaly without other brain abnormalities at birth.

## Conclusions

In summary, based on this multi-center study in an area not yet experiencing, but at risk of, Zika outbreaks, the estimated prevalence of microcephaly was higher than expected. Four factors, namely maternal HBV carrier status, primiparity, SGA, and preterm birth, are associated with 66.4% of the increased risk of microcephaly, and the risk factors varied between different subtypes of microcephaly. This study contributed to the better understanding of microcephaly subtypes characteristics. In addition, as infection is a main cause of microcephaly, the association between maternal HBV carrier status and microcephaly, which was first reported in this study, warrants further investigations.

## Data Availability

The data that support the findings of this study are not shared due to national and regional data regulation policy.

## Abbrevations

HC: Head circumference
GWCMC: Guangzhou Women and Children’s Medical Center
CNS: Central nervous system
GDM: Gestational diabetes mellitus
HDP: Hypertensive disorders of pregnancy
HBV: Hepatitis B virus
SGA: Small for gestational age
OR: Odds ratio
CI: Confidence interval
PAF: Population attributable fraction
